# Study on Sociodemographic Profiles and Health-Seeking Behaviour in Cervical Cancer Patients in a Tertiary Healthcare Centre in Central India

**DOI:** 10.7759/cureus.48635

**Published:** 2023-11-10

**Authors:** Angelin Priya, Padma Bhatia, Nisha Singh

**Affiliations:** 1 Community Medicine, Manipal-Tata Medical College Jamshedpur, Manipal Academy of Higher Education, Jamshedpur, IND; 2 Community Medicine, Gandhi Medical College, Bhopal, Bhopal, IND; 3 Community Medicine, Index Medical College, Hospital & Research Center, Indore, IND

**Keywords:** socio-demographic, india, health seeking behaviour, cancer cervical, cancer cervix

## Abstract

Introduction: Cervical cancer is one of the fourth most common cancers in females. Although screening and early intervention are preventive and a part of national programs, cervical cancer is attributed to a large number of deaths due to late-stage presentation and late diagnosis. To better understand this phenomenon, this study analyzed the sociodemographic characteristics of cervical cancer patients and assessed their health-seeking behaviour.

Methods: This cross-sectional study included 230 cervical cancer patients from the cancer ward of a tertiary care hospital. Detailed information about the onset of symptoms and previous gynecological problems along with sociodemographic details were recorded.

Results: Of the 230 cervical patients included, 70% were from rural areas and the majority presented at Stage III and IV. Additionally, 173 out of 230 patients previously had gynaecological problems, of which more than 50% did not initially seek any treatment. The common reasons for not seeking treatment were embarrassment, loss of daily wages, and the thought that it would heal by itself. The majority of those who took treatment went to traditional healers, and a Papanicolaou smear was not conducted on any patient.

Conclusion: Lack of awareness about the importance of screening programs along with the embarrassment of addressing any problems leads to unwillingness to access health care for cervical health.

## Introduction

Advances in medical technology have not reduced the deaths caused by cancer worldwide [1,2]. The global cancer mortality rate for cancers are higher than that of HIV/AIDS, tuberculosis, and malaria combined [3,4]. Cervical cancer is the fourth common cause of cancer globally according to the WHO, and approximately 604,000 new cases were identified in 2020, leading to 342,000 deaths [5]. Since cervical cancer is characterized by a long disease course from pre-invasive to invasive cancer, incidence of invasive cancer can be decreased by the early detection of pre-invasive and early stages. The pre-invasive stage can be detected through cytological screening (e.g., a Papanicolaou [Pap] smear). Screening enables the detection of curable premalignant lesions. In developed countries, a reduction in morbidity and mortality has been attained by the introduction of Pap smears, as the proportion of women screened is high. Screening coverage is low in Asian countries and India’s screening coverage is even lower. The Pap smear screening method requires numerous resources like histopathological setups, equipment, trained personnel, and transport of specimens. In developing countries like India, with a lack of such resources along with other public health priorities and lack of awareness, it becomes almost impractical to reach the population through cytology-based screening programs. As a result, the diagnosis of cervical cancer usually occurs after the onset of symptoms [3,6-9].

Despite the long latent and preinvasive phase, more than 85% of the patients present advanced stages [10]. The reason behind this delay could be personal and socio-demographic factors that may define health-seeking behaviour. The knowledge of these factors could be pivotal in building alternative strategies for the prevention of disease progression [11]. Thus, we conducted this study to analyze the sociodemographic profile and health-seeking behavior of patients diagnosed with cervical cancer in a tertiary care hospital.

## Materials and methods

This was a cross-sectional study conducted on patients with cervical cancer visiting the Department of Radiotherapy for chemotherapy or radiation therapy at a tertiary care hospital in central India. Sample size was calculated on the basis of a pilot study done for two weeks. All patients with cervical cancer reporting to the cancer outpatient department more than 18 years of age were included. Both new patients and follow-up patients were included in this study. Those who did not give consent to participate and those severely ill were excluded. After obtaining informed consent, all patients were interviewed, and details were recorded on a predesigned, pretested, and semi-structured questionnaire. The questionnaire included history regarding sociodemographic variables, menstrual history, family history of any cancer, and information regarding smoking, including anyone smoking in the household (recorded as passive smoking). Sexual and obstetric history, including the number of children (parity), birth spacing, and age at pregnancy were also recorded. Detailed information regarding the type of presenting symptoms that led to a cancer diagnosis was recorded along with the stage of cancer at which the diagnosis was made, histopathological type of the cancer, and any current treatment. To understand the health-seeking behaviour, the history of previous illness or any gynecological symptoms three years prior to the cancer symptoms were recorded. Detailed information about any treatment sought during that time and, type of health provider consulted was also recorded. Reasons for not seeking treatment were noted from those who had not taken any treatment earlier. All information was documented in the questionnaire.

All data were entered in Excel® (Microsoft, Redmond, WA, USA), and relevant statistical tests of significance like Chi-square test were applied wherever necessary. The categorical variables were presented in tabular form as frequency and percentage, while association between two categorical variables was determined using Chi-square test. Statistical significance of association was considered if the value of p was less than 0.05. To understand the factors behind not seeking treatment, sociodemographic variables were cross-tabulated with those who sought treatment and those who did not. Ethical clearance was obtained by the Institutional Ethics Committee of Gandhi Medical College (approval 138727-28/MC/IEC/2015).

## Results

Table 1 shows the distribution of participants according to sociodemographics and Table 2 shows the distribution of participants according to presenting symptoms.

**Table 1:** Distribution according to baseline variables of the study participants

Characteristics		Frequency (n = 230)	Percentage (%)
Age group	30–39	37	16.08
	40–49	75	32.60
	50–59	56	24.34
	60–69	50	21.73
	>70	14	06.00
Religion	Hindu	206	89.56
	Muslim	24	10.43
Locality	Rural	161	70
	Urban	69	30
Marital status	Married	200	86.95
	Unmarried	0	0
	Widow/Separate	30	13.04
Family history of cervical or any cancer	Yes	0	0
	No	230	100
Age at first pregnancy	<18 years	73	31.73
	18-21 years	125	54.34
	>21 years	32	13.91
Parity	<3	27	11.73
	3-6	152	66.08
	>6	51	22.17
Birth spacing	<2 years	71	30.86
	>2 years	159	69.13
History of intercourse with more than one partner	Yes	9	03.91
	No	221	96.08

**Table 2:** Distribution of patients according to symptoms and stage during diagnosis

Characteristics		Frequency	Percentage(%)
Presenting symptom	Post-menopausal bleeding	106	46.08
	Vaginal discharge	49	21.30
	Intermenstrual bleeding	47	20.43
	Post-coital bleeding	11	04.78
	Abdominal pain	15	06.52
	Burning micturition	2	00.86
Stage at diagnosis	I	06	02.60
	II	79	34.34
	III	106	46.08
	IV	39	16.95
Histological type	Squamous cell carcinoma	221	96.08
	Adenomatous carcinoma	06	2.60
	Epidermoid	02	00.86
	Papillary squamo transitional	01	00.43
Treatment	Combined chemotherapy and radiation therapy	165	71.73
	Chemotherapy only	08	03.47
	Radiation therapy only	37	16.08
	Combined therapy after surgery	20	08.69

Out of 230 participants, 173 (75.22%) had a history of gynecological symptoms in the last three years, while 57 (24.78%) did not and came for the first time with complaints of gynecological symptoms. Of these 173 participants with previous gynecological symptoms, only 83 (47.97%) sought treatment, while 90 (52.02%) did not take any treatment. The majority of participants (48.19%) first visited private practitioners, followed by traditional healers (26.50%), while very few reported going to a government primary-level or tertiary-level facility. Only 5.21% of participants received a Pap smear, whereas 94.78% never had a Pap smear. Of the 90 patients who did not seek treatment, 45% cited they did not recognize the seriousness of the symptoms. Other reasons cited for not seeking treatment were being busy (19%) and fear of loss of daily wages (17%). A few participants (11%) also cited they were too embarrassed to disclose symptoms and that the health facility was too far. Table 3 shows the distribution of patients who had received previous treatment and those who had not as per their sociodemographic characteristics. In the present study, a significantly higher proportion of patients residing in rural areas did not seek treatment (p < 0.05). No significant association of treatment-seeking behavior with age, education, and symptoms (p > 0.05) was found.

**Table 3:** Characteristics of participants according to their health-seeking behaviour

Characteristics	Treated n = 83 (%)		Not Treated n = 90 (%)	P-value
Age				
<60 years	60 (72.22)		67 (74.44)	0.75
>60 years	23 (27.77)		23 (25.55)	
Education				
No education	54 (65.06)		67 (74.4)	0.23
<5 years of school	17 (20.48)		10 (11.1)	
>5 years of school	12 (14.5)		13 (14.44)	
Locality				
Rural	51 (61.44)		74 (82.22)	0.002
Urban	32 (37.34)		16 (17.77)	
Symptoms				
Abnormal vaginal discharge	58 (69.87)		57 (63.33)	0.89
Genital itching	4 (4.8)		6 (6.66)	
Post-coital bleeding	14 (16.8)		17 (18.88)	
Others and intermenstrual bleeding	7 (8.4)		7 (7.77)	
Pap smear test ever done				
Yes	12 (14.45)		0	NA
No	71 (85.5)		90 (100)	
Pap smear test done	Government	Private		
Yes	05 (23.80)	7 (11.29)		0.16
No	16 (76.19)	55 (88.70)		

## Discussion

In our study, the mean age of participants was 50 ± 11.68 years, which was similar to previous studies in Tehran by Mosha et al. [12], in Tanzania by Torkzahrani et al. [13], and in Delhi by Rajaram et al. [14] with mean ages of 51, 57.8 ± 11.02, and 50.1 years, respectively. Our study included a majority of patients (70%) from rural areas, with only 30% from urban areas. Similar demographics were seen in a study done in Assam by Paul et al., [15] and in Mysuru Karnataka by Kaverappa et al., [16] in which 80% and 73.9% of the participants were from rural areas, respectively. The higher percentage of patients from rural areas may be due to a lack of access to healthcare facilities in these areas causing these patients to seek treatment elsewhere. This study showed about 86.95% of the study participants were married and the rest were either separated or widowed (13.04%). None of the participants were unmarried, which is similar to another study by Rajarao et al. [17]. Additionally, a study in Tanzania by Mosha et al. (2009) found that only 3% of the participants were single, while the rest were either married once or were co-habiting. In this study, none of the participants had a family history of cervical cancer. However, it is possible that cervical cancer was undetected in the earlier generation due to the lack of awareness and lack of access to health facilities. No Indian study could be identified showing any relationship between family history and cervical cancer. In the present study, almost 71.30% of the participants had no education, and only 18.69% had any education of any kind. These demographics were similar to other studies in which the majority of the patients were illiterate (Kaku et al. [8], Rajarao et al. [17], Torkzahrani et al. [13] and Kaverappa et al. [16]). The lack of education may contribute to the lack of awareness regarding early symptoms and the embarrassment in addressing gynecological problems.

In this study, the majority of the participants belonged to Class III (32.75%) & IV (38.86%) of the Modified B.G Prasad Classification of socioeconomic status. The classifications are slightly dissimilar to a study by Kaverappa et al. in which the majority (34.3%) of the patients belonged to Class III. Similar findings were seen in other studies, in which the majority of the participants belonged to a low socioeconomic class as in Paul et al. [15], Rajaram et al. [14], and Parikh et al. 2003 [18]. Also, in the meta-analysis, Parikh et al. [18] found an increased relative risk of dysplasia and cervical cancer with decreasing social class. History of sexual intercourse with more than one partner and having had more than two lifetime sexual partners have been shown to be significant risk factors for invasive cervical cancer in Franceshi et al. [19]. However, a history of sexual intercourse with more than one partner was only reported in 03.91% of the study participants in this study. A study by Tanturovski et al. [20] in Macedonia found that the vast majority of patients belonging to both early- and late-stage presentation of cervical cancer reported having two or fewer lifetime sexual partners (80% and 75.32%, respectively). This discrepancy might be due to the fact that in a country like India, having sex outside marriage or before marriage is considered taboo, so participants may have not revealed the information regarding sexual partners. The habit of chewing and smoking tobacco in the form of Beedi was seen in 10% and 7.39% of the participants, respectively, which may be a predisposing factor for the disease occurrence. Appleby et al. [21] found that the risk of squamous cell carcinoma is high in current smokers and increases with the number of cigarettes smoked per day and the duration of smoking. However, an Indian study by Kaverappa et al. found that survival was poor in patients with cervical cancer who chewed tobacco. Passive smoking was seen in the majority of our participants (53.04%). However, in a study by Louie et al. [22] passive smoking was not found as an independent risk factor of invasive cervical cancer. On the contrary, a study by Zeng et al. [23] found a 73% increase in the risk of cervical cancer in those who never smoked but experienced smoking. This factor needs to be further investigated.

Out of 230 total participants and 173 (75.21%) participants with previous gynecological symptoms, only 47.97% sought treatment. The majority of participants (48.19%) visited private practitioners, followed by traditional healers (26.50). Gyenwali et al. [24] found that ignoring the mild gynecological symptoms as well as dependency on traditional healthcare practices were the reasons for not seeking treatment early.

The main reason for not seeking treatment in the present study was not recognizing the seriousness of the symptoms. Some participants were too busy to go to a healthcare provider, where 16.66% found that going to the doctor would take a lot of their time leading to a loss of daily wages. Others (11.11%) were hesitant to disclose the symptoms to the doctor and 7.77% did not seek any treatment since the health facility was distant. Similar results were found in a study by Forbes et al. [25] where patients cited lack of awareness of the seriousness of the symptoms, being too ashamed to see the doctor, worry about the diagnosis, and being too busy for delaying going to the doctor. Also, only 5.21% of the participants received a Pap smear, whereas 94.78% never had a Pap smear. These findings were consistent with a study by Langley and Mary [26] in which 49% knew about Pap smears but never got one. Our study found a significant association between patients who did not seek treatment and their locality. As mentioned by Parikh et al. the reason for this association must be the inaccessibility of health services and lack of awareness regarding such screening tests, which is seen in low socioeconomic classes [18]. The lack of health awareness can be attributed to GDP spent on health.

Our study had some limitations. This study included some follow-up patients who gave information based on their memory, which could have led to underreporting of any facts. High chances of recall bias can be there. Additionally, focus group discussions could have brought fresh perspectives from participants, which was not done in our study.

## Conclusions

The predominance of patients were from rural areas and low socioeconomic class with poor health-seeking behavior. It is imperative that awareness regarding cervical cancer, as well as mass screening campaigns, be prioritized for early detection and management of the disease. Intensive educational intervention is needed for mass awareness to emphasize the need to treat reproductive problems at the earliest. Such changes will bridge the gap between health inequity and ensure universal health coverage for all.
